# Staged Total Hip Arthroplasty for Septic Hip Following Core Decompression in Sickle Cell-Related Osteonecrosis: A Case Report and Review of the Literature

**DOI:** 10.7759/cureus.105124

**Published:** 2026-03-12

**Authors:** Mohammed Alenezi, Salamah Ayyad, Thunayan Alemairi

**Affiliations:** 1 Department of General Surgery, Al Adan Hospital, Kuwait City, KWT; 2 Department of Orthopedics, Jaber Al Ahmed Al Sabah Hospital, Kuwait City, KWT; 3 Department of Orthopedics, Al Razi Hospital, Kuwait City, KWT

**Keywords:** crp, onfh, osteonecrosis, scd, sickle cell disease

## Abstract

Osteonecrosis of the femoral head is a well-recognized musculoskeletal complication of sickle cell disease (SCD) that frequently results in early joint destruction and functional disability in young patients. The coexistence of avascular necrosis and septic arthritis in SCD patients is uncommon and presents significant diagnostic and therapeutic challenges. We report the case of a 24-year-old male patient with sickle cell disease who developed septic arthritis of the hip following femoral head core decompression, ultimately requiring staged total hip arthroplasty.

## Introduction

Osteonecrosis of the hip joint is a common and debilitating complication of sickle cell disease, frequently necessitating surgical intervention, often at a young age [1]. The pathophysiology involves vaso-occlusion secondary to sickled erythrocytes, which compromises blood flow and oxygen supply to the femoral head and precipitates bone infarction. Patients who are homozygous for the sickle cell gene (hemoglobin SS) are at increased risk of bone infections. This susceptibility is attributed to recurrent episodes of intestinal mucosal sloughing that predispose to enteric bacteremia in addition to osteonecrosis from microvascular occlusion.

Core decompression, which involves simple drilling of the femoral head to release intraosseous hypertension, can be considered for early-stage, uncollapsed lesions in younger patients; its efficacy in preventing progression to advanced disease remains limited, particularly in the context of subsequent septic arthritis [2,3]. This complex clinical scenario often progresses to advanced stages requiring total hip arthroplasty, which presents unique challenges in this patient population [4]. Total hip arthroplasty offers substantial long-term pain relief and improved function for these patients, although the decision for surgical intervention requires careful consideration due to the patient's age and underlying condition [4].

This consideration becomes even more critical when managing the sequelae of septic arthritis, which can still be effectively treated with total hip arthroplasty, often yielding superior functional results compared to alternative procedures like resection arthroplasty or arthrodesis [4,5]. This case report aims to highlight the successful management of a young patient with sickle cell disease who developed septic arthritis of the hip following core decompression, subsequently requiring total hip arthroplasty, and to discuss the unique challenges and outcomes associated with this complex clinical presentation.

## Case presentation

A 24-year-old male with a known history of sickle cell disease presented with severe left hip pain. His past surgical history included cholecystectomy, splenectomy, and four previous femoral head core decompression procedures performed at multiple institutions. Two months prior to presentation, he underwent surgical debridement and washout via an anterior approach for septic arthritis of the hip. The patient was referred to our institution while admitted elsewhere for a vaso-occlusive crisis and persistent left hip symptoms suggestive of ongoing infection.

Clinical examination revealed significant swelling, erythema, and tenderness over the left hip joint, accompanied by a restricted range of motion, limb length discrepancy, and a Harris Hip Score of 20 [6]. Laboratory investigations demonstrated leukocytosis with a white blood cell count of 14.4/L (3.7-11/L), elevated C-reactive protein of 65 mg/L (0-8 mg/L), and erythrocyte sedimentation rate of 70 mm/hr (0-20 mm/hr). Joint aspiration cultures yielded *Staphylococcus lugdunensis*.

**Figure 1:**
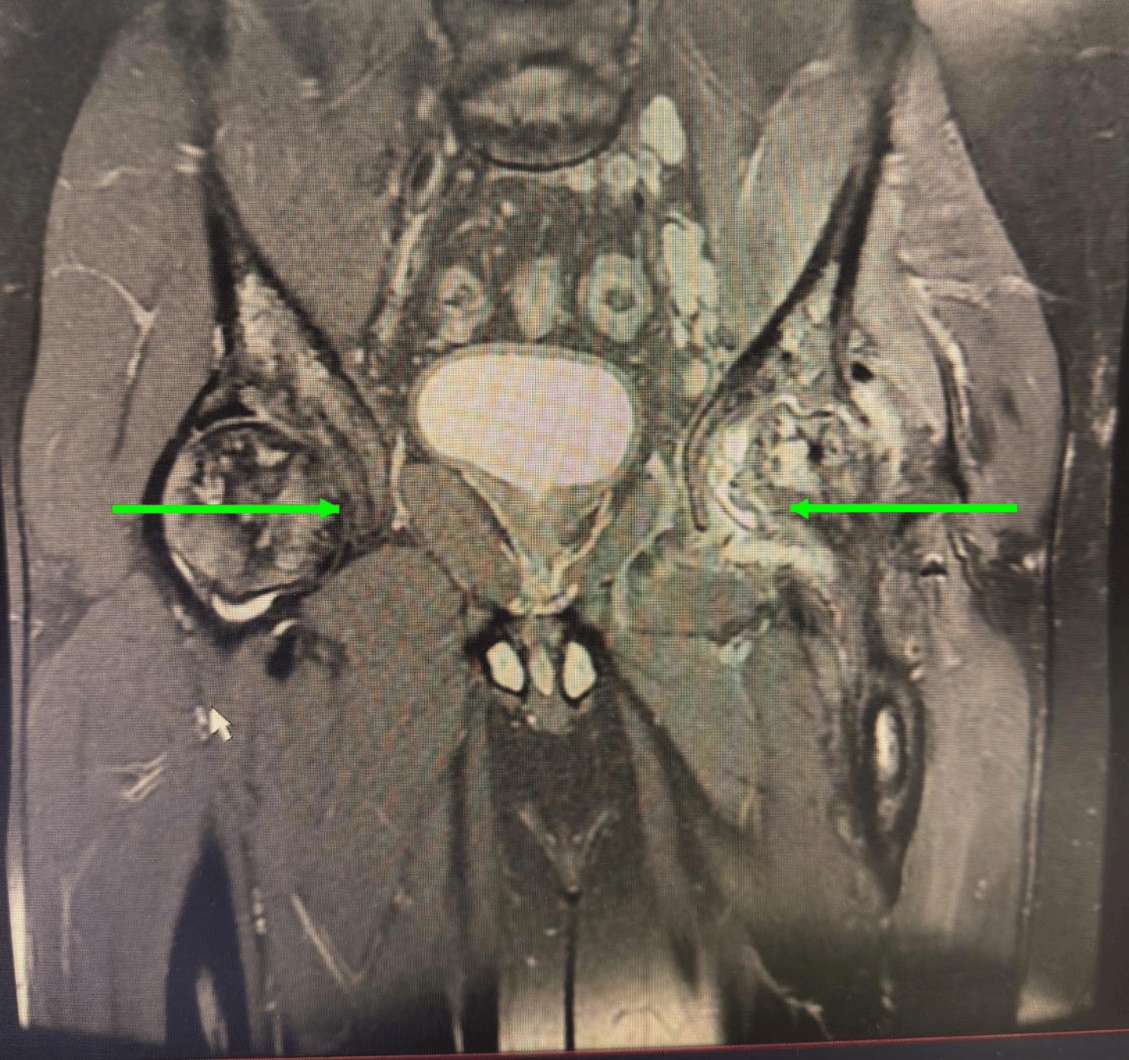
Contrast-enhanced magnetic resonance imaging showing bilateral femoral head osteonecrosis, with additional findings of left proximal femoral osteomyelitis and septic arthritis of the left hip associated with intraosseous and peri-osseous communicating abscess formation. The right green arrow shows left proximal femoral osteomyelitis and septic arthritis with associated intraosseous and peri-osseous communicating abscess formation, and the left green arrow shows right femoral head osteonecrosis.

Preoperative radiographs demonstrated advanced collapse of the femoral head with mixed sclerotic and lytic changes consistent with Ficat stage IV avascular necrosis. Contrast-enhanced magnetic resonance imaging confirmed bilateral femoral head osteonecrosis, with additional findings of left proximal femoral osteomyelitis and septic arthritis of the left hip associated with intraosseous and peri-osseous communicating abscess formation (Figure 1).

**Figure 2:**
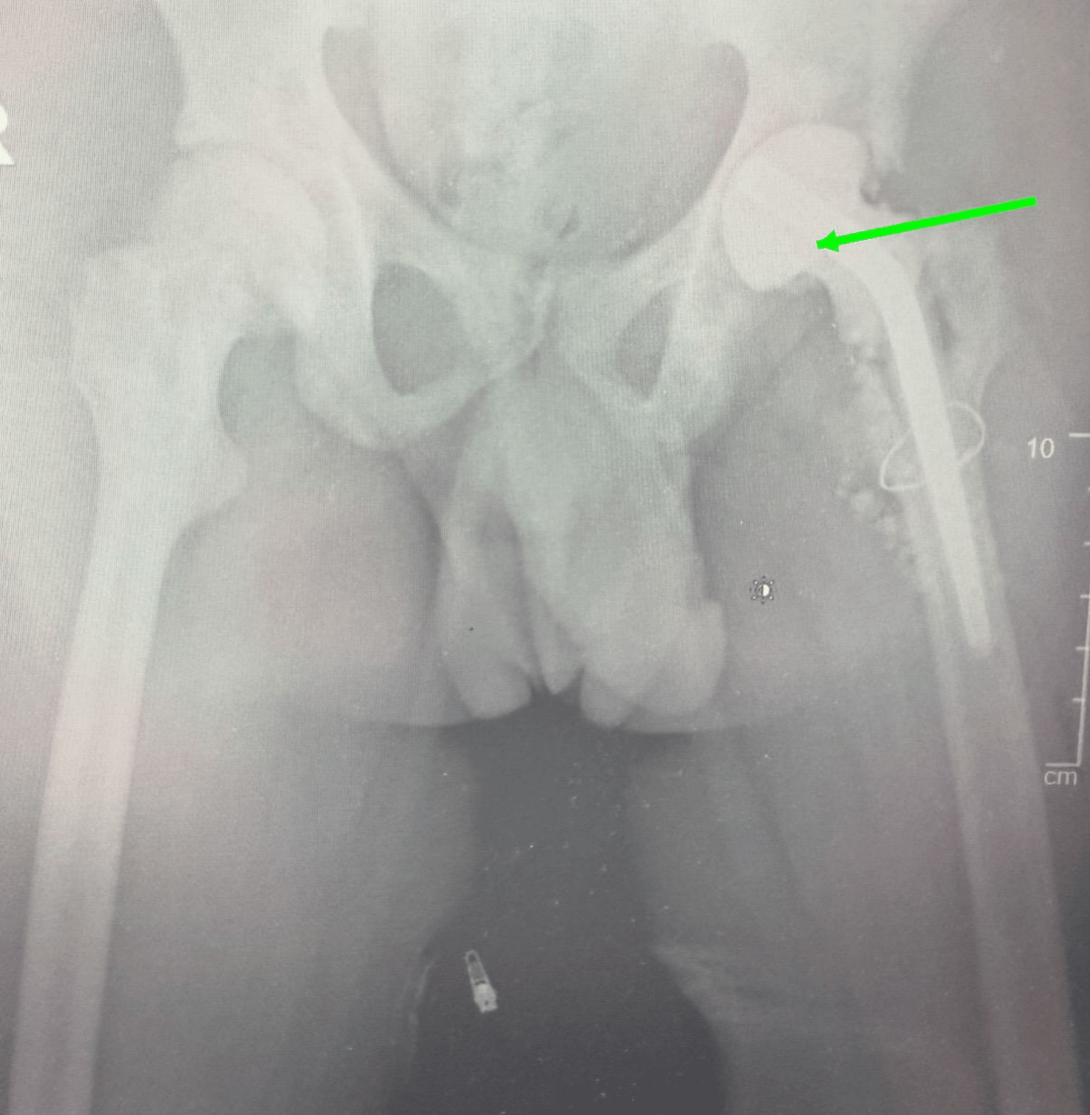
Cement spacer following first-stage THA Immediate postoperative X-ray of the pelvis following first-stage THA showing antibiotic loaded cement spacer implantation (green arrow). THA: total hip arthroplasty.

Given the chronic infection and complex underlying pathology, a staged total hip arthroplasty was planned. During the first stage, a posterior surgical approach was utilized with careful soft tissue handling. Intraoperatively, minimal purulence and extensive fibrotic tissue were encountered. Following femoral head osteotomy, the bone was noted to be markedly sclerotic. The femoral canal was thoroughly debrided and sequentially prepared using revision rasps. After extensive irrigation, an antibiotic-loaded cement spacer was inserted (Figure 2).

**Figure 3:**
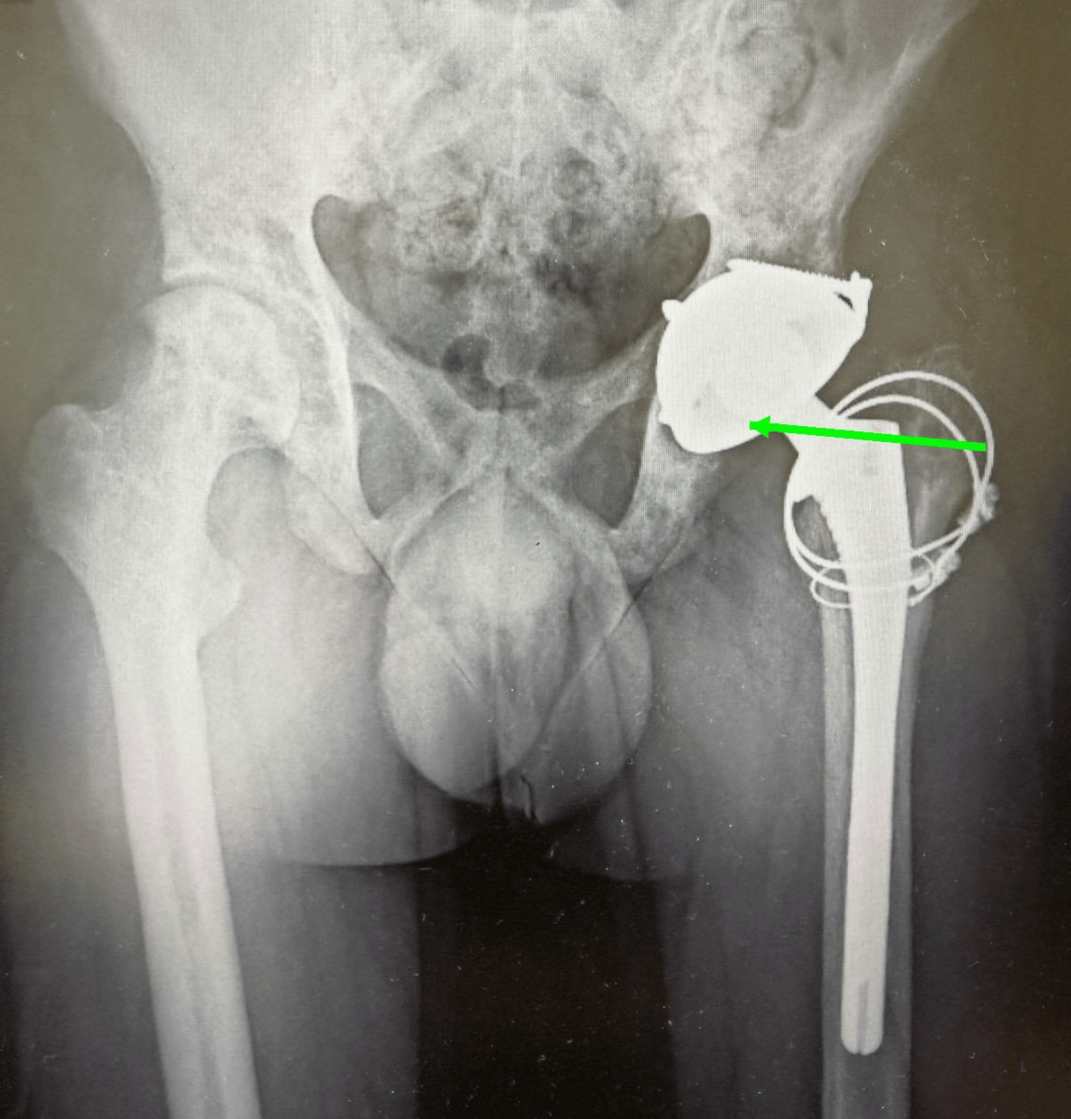
Immediate postoperative X-ray following second-stage THA Uncemented dual mobility implant following second-stage THA (green arrow). THA: total hip arthroplasty.

A minor intraoperative femoral fracture occurred during spacer insertion, which was successfully stabilized using cerclage wiring. The wound was closed in layers. Postoperatively, the patient remained hospitalized for close monitoring. Intraoperative tissue cultures subsequently grew *Staphylococcus capitis*. A multidisciplinary team including infectious disease specialists guided targeted intravenous vancomycin and gentamycin therapy for six weeks. Serial inflammatory markers and repeat cultures confirmed eradication of infection. Following normalization of inflammatory parameters and negative culture results after two weeks off the antibiotic course, the patient underwent the second-stage reconstruction. The cement spacer was removed, and an uncemented dual-mobility total hip arthroplasty was performed (Figure 3).

An uncemented implant was selected considering the patient’s young age and anticipated need for future revision procedures. Additional cerclage wires were applied to enhance femoral stability. The procedure was completed without complications, and intraoperative assessment confirmed restoration of equal limb lengths.

**Figure 4:**
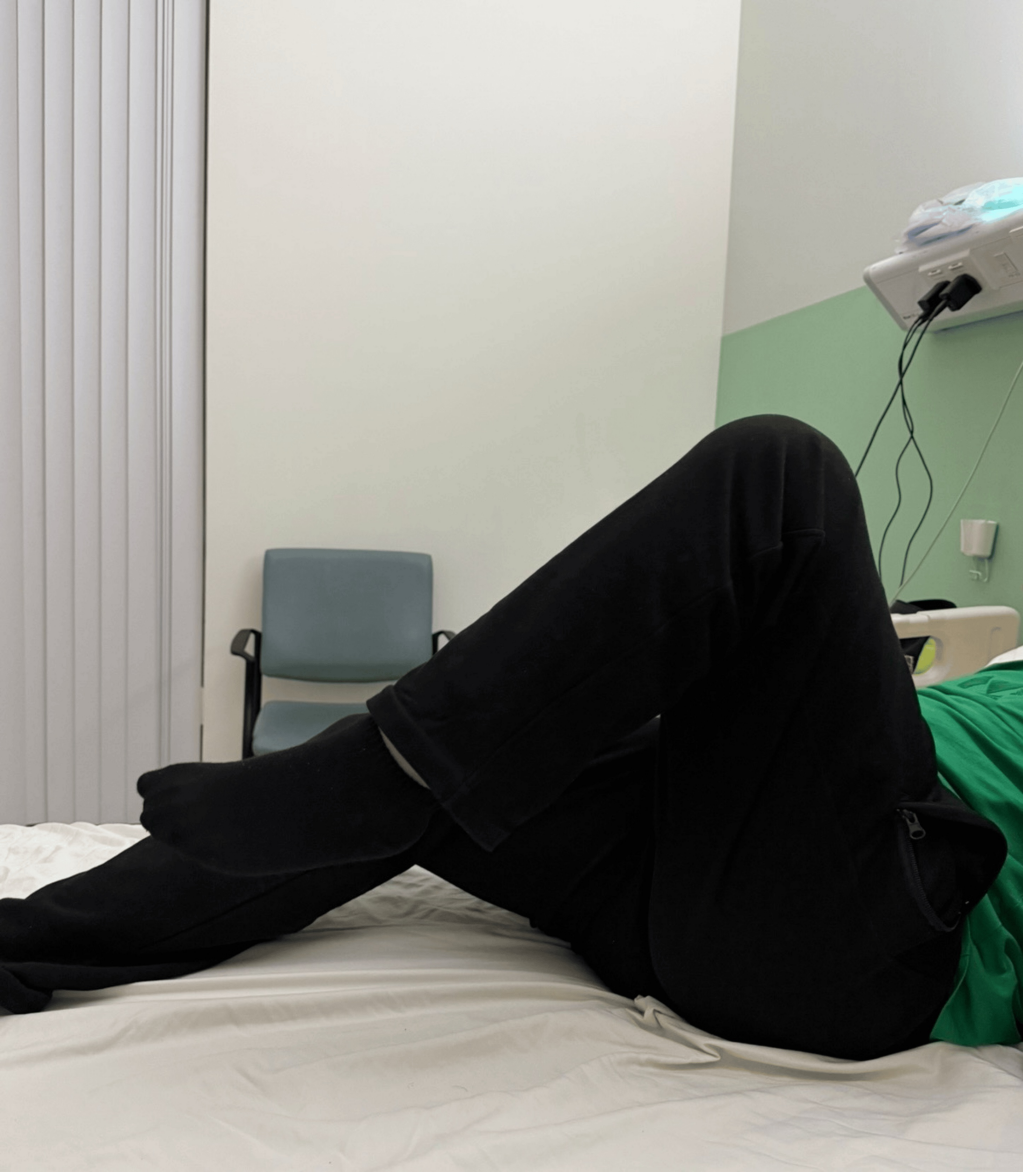
Left hip flexion. Post-THA range of motion at 12-month follow-up. THA: Total Hip Arthroplasty.

**Figure 5:**
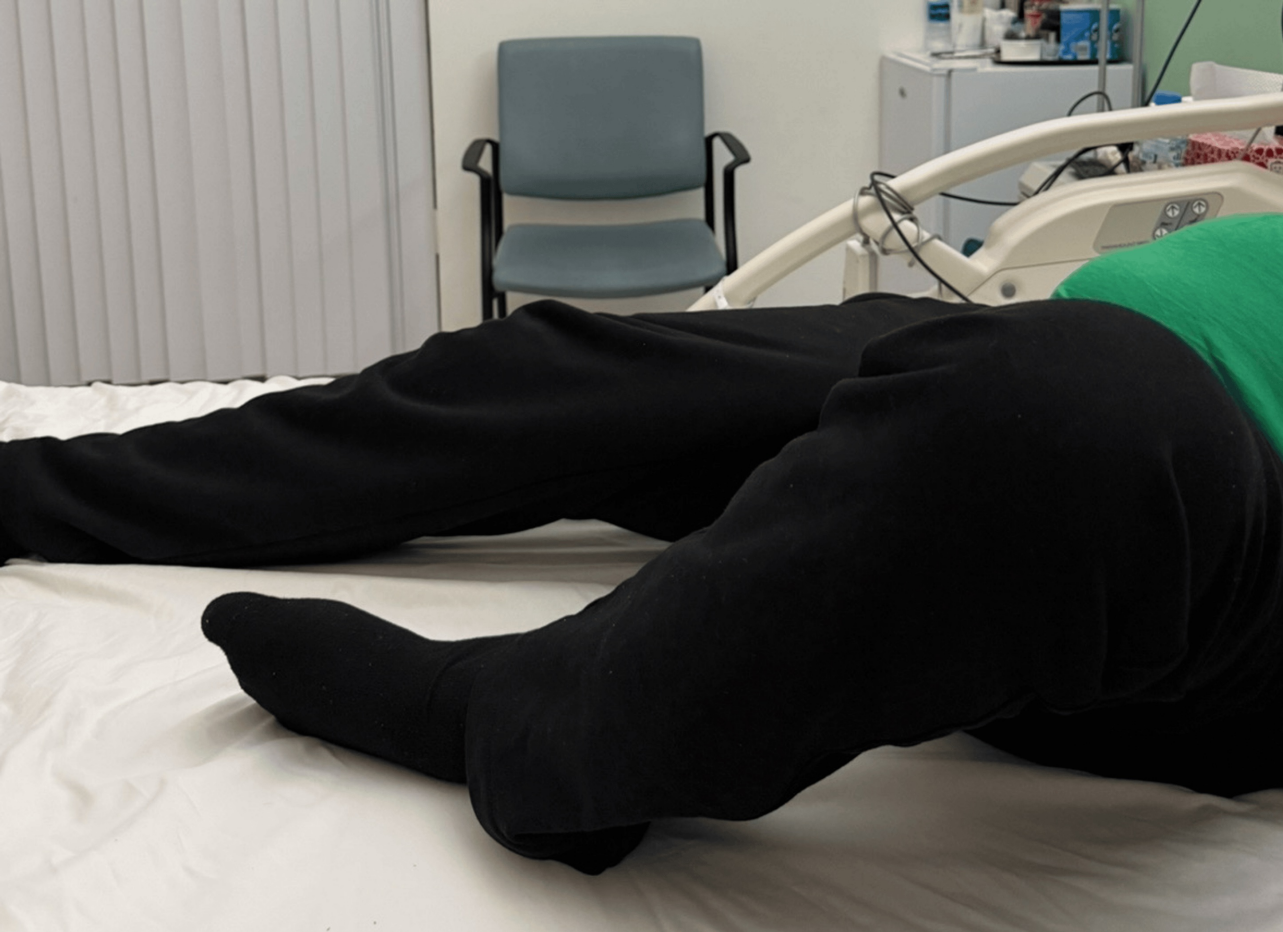
Left hip abduction.

The postoperative course was uneventful, with gradual improvement in pain and functional mobility. However, eight months following surgery, the patient presented to another institution with ipsilateral knee swelling, pain, and erythema. Joint aspiration confirmed *Escherichia coli* infection. The patient underwent knee arthrotomy and washout, which resulted in complete resolution of symptoms. At 12-month follow-up, the patient demonstrated full hip range of motion, symmetrical limb lengths, and a Harris Hip Score of 94. He was able to perform activities of daily living without limitation, including sitting cross-legged comfortably (Figures 4, 5).

The patient currently remains clinically stable and continues routine follow-up while awaiting bone marrow transplantation.

## Discussion

Osteonecrosis of the femoral head (ONFH) is one of the most debilitating complications of sickle cell disease, often leading to premature joint destruction and necessitating surgical intervention. The ischemic insult creates a vulnerable environment for bacterial colonization, particularly following invasive procedures such as core decompression, which can serve as a portal of entry for pathogens and subsequently precipitate septic arthritis [5]. While femoral head osteonecrosis (ONFH) is common in this population, the occurrence of superimposed septic arthritis is considerably rare and is often underdiagnosed because symptoms may overlap and be masked by the chronic pain. Previous studies have collectively reported 44 patients with osteonecrosis of the femoral head complicated by septic hip arthritis (Table 1). To the best of our knowledge, this represents the first reported case of septic arthritis of the hip developing after core decompression performed for osteonecrosis in a patient with sickle cell disease, ultimately necessitating total hip arthroplasty.

**Table 1:** Previously reported patients with osteonecrosis of the femoral head (ONFH) accompanied by septic hip. a: Bilateral ONFH. b: Bilateral septic arthritis. c: Previous use of antibiotics. ONFH: osteonecrosis of the femoral head; PMN: polymorphonuclear leukocytes; ESR: erythrocyte sedimentation rate; CRP: C-reactive protein; SLE: systemic lupus erythematosus; CS: corticosteroid use; TPL: transplantation; HD: hemodialysis; MRI: magnetic resonance imaging; MSSA: methicillin-sensitive *Staphylococcus aureus*; MRCNS: methicillin-resistant coagulase-negative staphylococci; M: male; F: female; (+): positive; (-): negative; NA: not available.

Study	Sex/Age	Risk factor of ONFH	Risk factor of septic arthritis	Fever	WBC count (% PMN)	ESR (mm/h)	CRP (>0.5 mg/dL)	Joint fluid WBC (% PMN)	MRI (infection)	Culture of joint fluid	Treatment
Galindo et al. (2005) [7]	F/47	CS with SLE	CS with SLE	+	NA	NA	NA	NA	+	MSSA	Antibiotics
Galindo et al. (2005) [7]	F^ab^/38	CS with SLE	CS with SLE	+	NA	NA	NA	NA	+	MSSA	Arthrotomy
Ostrum (1993) [8]	F/51	CS for heart TPL	Nocardia pneumonia	+	10,400 (90%)	NA	NA	NA	NA	Nocardia^c^	Arthrotomy
Nuovo et al. (1991) [9]	M^c^/45	Alcohol-related	Intravenous drug abuse	-	4,000 (47%)	36	NA	NA	NA	Staphylococcus aureus^c^	Arthrotomy
Phillips and Pottenger (1988) [10]	M^a^/32	CS for HD	Subacute endocarditis	+	12,800	67	NA	NA	NA	Streptococcus viridans	Arthrotomy
Phillips and Pottenger (1988) [10]	M^a^/53	Idiopathic	None	-	6,300	95	NA	NA	NA	S. aureus	Arthrotomy
Phillips and Pottenger (1988) [10]	M/14	Sickle cell disease	Pharyngitis	+	17,700	NA	NA	20,000	NA	Haemophilus influenzae	Arthrotomy
Phillips and Pottenger (1988) [10]	M^a^/33	CS for renal TPL	Pharyngitis	-	18,700	NA	NA	NA	NA	Streptococcus	Arthrotomy
Shiota et al. (1981) [11]	F/36	CS with SLE	GI salmonellosis	+	12,500	127	NA	NA	NA	Salmonella typhimurium^c^	Arthrotomy
Hauermann and Friedenthal (1978) [12]	F/25	CS for renal TPL	Deep infection after TPL	+	NA	NA	NA	NA	NA	S. aureus^c^	Repeated aspiration
Hauermann and Friedenthal (1978) [12]	F/21	CS with SLE	Skin infection	+	NA	NA	NA	NA	NA	Negative^c^	Repeated aspiration
Hauermann and Friedenthal (1978) [12]	M^ab^/30	CS for renal TPL	Salmonella sepsis	+	NA	NA	NA	70,000; 30,000	NA	Negative^c^; *Salmonella^c^*	Repeated aspiration
Hauermann and Friedenthal (1978) [12]	M/20	CS for renal TPL	None	+	NA	NA	NA	24,000	NA	Peptostreptococcus	Repeated aspiration
Lee et al. (2011) [13]	M/36	Alcohol-related	None	-	4,280 (49%)	29	3.35	110,000 (89%)	NA	Negative	Arthroscopy
Lee et al. (2011) [13]	M^a^/75	Alcohol-related	None	-	7,260 (72%)	53	3.15	NA	+	Negative	Arthroscopy
Lee et al. (2011) [13]	M^a^/48	Alcohol-related	None	-	6,120 (63%)	19	5.39	NA	NA	Negative	Arthrotomy
Lee et al. (2011) [13]	M^a^/51	Alcohol-related	Osteomyelitis of toe	+	12,770 (46%)	18	1.13	23,000 (86%)	+	Negative^c^	Arthroscopy
Lee et al. (2011) [13]	M^a^/37	Steroid-related	Subcutaneous abscess	+	7,090 (68%)	23	4.12	195,000 (96%)	+	MRCNS^c^	Arthroscopy
Kim et al. (2021) [14]	M/50	Alcohol-related	Alcoholic liver cirrhosis; end-stage renal disease	+	13,100	76	7.21	NA	+	Stenotrophomonas maltophilia^c^	Arthroscopy
Kim et al. (2021) [14]	F/44	Idiopathic	None	+	NA	62	14.7	400,000 (73%)	+	S. aureus^c^	Arthroscopy
Lee et al. (2019) [15]	14 patients	NA	None	3 patients+	NA	All patients had elevated serum ESR (>20 mm/h) and/or CRP (>0.5 mg/dL)	All patients had elevated serum ESR (>20 mm/h) and/or CRP (>0.5 mg/dL)	NA	12 patients+	NA	Two-stage re-implantation
Li et al. (2024) [16]	F/67	Alcohol-related	None	-	5.26 (83.50)	39.7	52.25	NA	+	Corynebacterium	Two-stage re-implantation
Li et al. (2024) [16]	M/76	Alcohol-related	None	-	6.8 (58.10)	19.5	51.69	NA	+	Negative	Two-stage re-implantation
Li et al. (2024) [16]	F/60	Alcohol-related	None	-	9.86 (69.30)	29.4	9.86	NA	+	MSSA	Two-stage re-implantation
Li et al. (2024) [16]	M/55	Alcohol-related	None	-	8.93 (86.50)	50.6	154.33	NA	+	MSSA	Two-stage re-implantation
Li et al. (2024) [16]	F/81	Idiopathic	None	-	7.04 (57.90)	56.3	30.3	NA	-	Negative	Two-stage re-implantation
Li et al. (2024) [16]	M/67	Alcohol-related	None	-	7.67 (62.50)	58	31.72	NA	-	Salmonella	Two-stage re-implantation
Li et al. (2024) [16]	M/74	Alcohol-related	None	-	17.29 (83.80)	20.2	48.6	NA	+	Citrobacter	Two-stage re-implantation
Li et al. (2024) [16]	M/68	Steroid-related	None	-	16.5 (68.14)	42.3	46.46	NA	+	Negative	Two-stage re-implantation
Li et al. (2024) [16]	F/70	Steroid-related	Nephrotic syndrome	-	8.39 (78.30)	11.4	165	NA	+	Salmonella	Two-stage re-implantation
Li et al. (2024) [16]	M/64	Idiopathic	None	-	13.4 (81.1)	10.2	194.77	NA	+	Klebsiella	Two-stage re-implantation
In this study	M/24	Sickle cell disease	Core decompression	-	14.4 (NA)	70	65	NA	+	Staphylococcus lugdunensis	Two-stage re-implantation

WBC, erythrocyte sedimentation rate (ESR), and C-reactive protein are standard preoperative laboratory tests prior to total joint arthroplasty and constitute key criteria for diagnosing periprosthetic joint infection [5]. ESR and CRP levels have been consistently described as early indicators of septic involvement even in the absence of fever or leukocytosis. Previous literature reported a total of 31 patients with ONFH accompanied by septic hip, all of whom had elevated ESR and/or CRP. Previous cases reported 23 patients, nine of whom had elevated white blood cell counts [16], suggesting that WBC count has limited diagnostic value for septic hip arthritis, whereas ESR and CRP demonstrate high specificity.

Plain radiographs represent the first-line imaging modality for assessing a suspected septic joint. Characteristic findings on radiography, including soft tissue swelling, joint space narrowing, periarticular osteopenia, and central or peripheral osseous erosions, typically emerge late following the onset of clinical infection. Li et al. reported nine patients in Ficat stage IV and one patient in Ficat stage III [16]. The advanced stage of osteonecrosis observed in these cases underscores the aggressive nature of the disease process and highlights the limitations of plain radiography in detecting early infectious changes before significant structural damage has occurred. Consequently, advanced imaging modalities are often required to facilitate early diagnosis and accurate assessment of the extent of osseous and soft tissue involvement. MRI offers high sensitivity for diagnosing musculoskeletal infections and provides a detailed assessment of osseous and soft tissue involvement. In septic arthritis, MRI typically reveals joint effusion, bone marrow edema, synovial thickening, and cartilage destruction. These advanced imaging characteristics are crucial for early detection and differentiation of septic arthritis from avascular necrosis alone, as timely identification is essential to prevent irreversible joint damage and guide appropriate surgical management. Prior studies have documented a total of 34 cases of osteonecrosis of the femoral head complicated by septic hip arthritis. Of these, 31 patients demonstrated positive MRI findings, including two who did not undergo preoperative MRI [16]. The high diagnostic yield observed in these cases underscores the utility of MRI in confirming the presence of infection and delineating the extent of pathology when plain radiographs are inconclusive.

Microbiological cultures serve not only to confirm the presence of infection but also to ascertain antibiotic susceptibility and resistance profiles. Previous studies have reported a total of 30 patients, 21 of whom yielded positive bacterial cultures. The comparatively low culture positivity rate may be attributed to antibiotic therapy prior to admission, delays in specimen processing, and insufficient incubation periods. Moreover, procuring an adequate volume of synovial fluid for microbiological evaluation remains technically challenging due to the "dry tap" phenomenon, thereby constraining the performance of preoperative cultures via conventional approaches [16].

Symptomatic femoral head osteonecrosis (ONFH) can be managed using the following surgical procedures: core decompression, rotational osteotomy, vascularized free fibular transfer, total hip replacement, total hip resurfacing, and hip arthrodesis. Among these options, core decompression is typically favored for early-stage osteonecrosis to preserve the femoral head, whereas total hip arthroplasty remains the definitive treatment for advanced joint destruction or when salvage procedures fail [2].

Once septic arthritis develops, the clinical management becomes significantly more complex, necessitating prompt antibiotic therapy and often requiring surgical debridement to prevent irreversible cartilage destruction and systemic sepsis. Previous literature strongly supports staged total hip arthroplasty (THA) in these cases, which involves an initial period of aggressive infection control through debridement and antibiotic administration prior to definitive joint reconstruction. This staged approach is critical in the sickle cell population to minimize the risk of periprosthetic infection, which can be catastrophic given the already compromised immune status and vaso-occlusive tendencies inherent to the disease. Studies evaluating ONFH with septic hip have demonstrated satisfactory clinical results following two-stage THA with no recurrence of infection during mid-term follow-up [16]. Similarly, case series have reported successful delayed arthroplasty performed several months after infection eradication with significant improvement in hip function [4]. Despite these encouraging outcomes, performing total hip arthroplasty in young patients with sickle cell disease presents unique challenges, including the potential for increased rates of perioperative complications, aseptic loosening, and the need for future revision surgery due to high functional demands and extended life expectancy. In this case, intramedullary reaming was utilized to prepare the femoral canal. This technique is particularly valuable in selected patient populations in whom the femoral canal becomes sclerotic, rendering conventional rasping insufficient for adequate canal preparation. In such situations, it is advisable to have Kirschner wires (DePuy Synthes, Warsaw, Indiana, USA) and intramedullary reamers available to help identify and gradually open the canal, thereby facilitating safe femoral stem insertion during total hip arthroplasty.

This consideration is especially relevant in patients with sickle cell disease, where repeated vaso-occlusive crises can result in endosteal bone formation, sclerosis, and narrowing or obliteration of the intramedullary canal [1]. In this patient, a dual mobility acetabular component was selected for several biomechanical and patient-specific reasons supported by existing literature. Dual mobility cups were originally designed to increase the effective femoral head diameter and improve hip stability, thereby reducing the risk of postoperative dislocation, particularly in high-risk patients. Studies have demonstrated significantly lower dislocation rates with dual mobility constructs compared with conventional bearings in both primary and revision total hip arthroplasty [17].

In our patient population, the acetabulum is frequently relatively shallow and smaller in morphology, which may compromise femoral head coverage and implant stability. Variations in acetabular geometry are known to influence press-fit stability and fixation of acetabular components, supporting implant selection strategies tailored to acetabular morphology. Additionally, in our case, intraoperative findings demonstrated abductor muscle weakness, a well-established risk factor for instability following total hip arthroplasty. Dual mobility constructs are specifically recommended in situations with compromised soft tissue envelope or abductor deficiency because they provide increased jump distance and range of motion, thereby enhancing stability and minimizing dislocation risk. Considering the combination of altered acetabular morphology and deficient abductor function, a dual mobility articulation was chosen to optimize acetabular coverage, improve biomechanical stability, and reduce the likelihood of postoperative instability.

As a single case report, the findings are constrained by limited generalizability and cannot establish definitive causality or statistical significance with respect to the optimal management strategy for this rare complication. Larger cohort studies with prospective data collection are warranted to better define the incidence, risk factors, and long-term outcomes of total hip arthroplasty following septic arthritis in patients with sickle cell disease.

## Conclusions

In conclusion, septic arthritis should be strongly suspected in patients with sickle cell disease presenting with worsening hip pain in the setting of osteonecrosis, especially post-core decompression procedures. Early diagnosis is best achieved by using elevated inflammatory markers in conjunction with magnetic resonance imaging. Prompt infection control through multidisciplinary management followed by staged arthroplasty with meticulous surgical technique and proper implant selection can result in favorable functional outcomes and improved quality of life.
